# Short-term outcomes of laparoscopic sleeve gastrectomy among obesity patients in the northern west bank: a retrospective records review

**DOI:** 10.1186/1756-0500-7-85

**Published:** 2014-02-07

**Authors:** Hamzeh Al Zabadi, Ahmad Daqour, Abdullah Hawari, Jihad Hasouni

**Affiliations:** 1 School of Medicine and Health Sciences-Public Health and Community Medicine Department, An-Najah National University, Nablus, Palestine; 2 School of Medicine and Health Sciences-Medicine Department, An-Najah National University, Nablus, Palestine; 3 Trauma and Surgical Department, Arab Specialized Hospital, Nablus, Palestine; 4 Surgical and Laparoscopic Department, Palestinian Red Crescent Society Hospital, Tulkarem, Palestine

## Abstract

**Background:**

Conservative methods for weight loss are usually disappointing. Therefore, surgeries such as Laparoscopic Sleeve Gastrectomy (LSG) should be considered. We aimed to evaluate the outcomes (body mass index; BMI) of LSG among obesity patients in the Northern West Bank.

**Methods:**

Hospital records were reviewed for all patients who had undergone LSG since 2010 in Arab specialized hospital in Nablus and Palestinian Red Crescent society hospital in Tulkarem. Then, patients have been invited again to participate in the study and asked to self-report further pre-/post-operative measures. The primary study outcome was the change in BMI while secondary outcomes included obesity associated co-morbidities’ measures; hypertension (HTN) and diabetes mellitus (DM).

**Results:**

The mean age (standard deviation; SD) of the study participants (n = 30; 20 women and 10 men) was 34.06 (10.71) years. The mean (SD) follow-up time was 7.16 (5.05) months. The mean ± SD of the pre-operative BMI was 47.23 ± 7.89 kg/m^2^ while 36.74 ± 7.74 kg/m^2^ post-operatively (95% CI for mean differences and *P-*value; 8.83-12.14 and 0.001). For the clinically diagnosed hypertensive patients, there was a mean (SD) reduction of 27.50 (9.87) mm Hg in systolic pressure (*P* < 0.026) and 18.33 (13.66) of the diastolic blood pressure (*P* < 0.042). For diabetics, there were clinically and biologically clear mean (SD) reductions in fasting blood sugar and glycated hemoglobin A1c of about 82.00 (22.70) mg/dl and 1.90 (0.78) %; respectively. Only practicing sports or exercise (no/yes) remained significant with post-operative BMI (regression coefficient B = −7.33; *P-*value and 95% CI for B; 0.009 and −12.68- -1.98).

**Conclusions:**

LSG can significantly improve BMI and could improve or resolve obesity associated co-morbidities like HTN and DM. LSG could be recommended for co-morbid obesity patients who fail to reach beneficial results from a structured weight loss programs.

## Background

Overweight and obesity are major public health problems. A person with a body mass index (BMI) ≥ 25 is considered as overweight while a person with a BMI of ≥ 30 is generally considered as obese [1]. In the past few decades, the incidence of obesity has raised dramatically in low and middle income countries, particularly in urban setting [1]. This emerges an increase in the incidence of diseases that are related to obesity such as hypertension, diabetes mellitus and cardiovascular diseases. These types of obesity co-morbidities have high risk of mortality and represent a heavy economical (both direct and indirect) burden on the patient himself from one hand and the primary health care system on the other hand [1]. Indeed, 44% of the diabetes burden, 23% of the ischemic heart disease burden and between 7% to 41% of certain cancers burdens are attributable to overweight and obesity [1].

In the year 2008, nearly 1.5 billion adults aged ≥ 20 years were overweight. Of them, approximately 200 million male and 300 million female were obese [1]. Moreover, it has been shown that overweight and obesity are the fifth leading risk for global deaths and that at least 2.8 million adults die each year as a result of being overweight or obese [1]. In Palestine, a study was conducted in the rural community in Ramallah city among the adults aged 30–65 years, the results showed that obesity and overweight were about 58.7% in men and 71.3% in women [2]. Another study showed that the prevalence of obesity in urban Palestinian population was 41% (49% and 30% for women and men; respectively) [3].

Therapeutic interventions for the treatment of obesity range from lifestyle and diet modifications to pharmacologic and surgical therapy [4]. However, studies showed that the non-operative interventions for sustained weight loss usually fail to provide real benefits and are usually insufficient and not sustainable in co-morbidity obese patients [5,6]. Now, the most popular weight loss surgery is the bariatric surgery. It is an operative surgery of the stomach and/or intestines that help a person with morbid obesity to lose weight. Of them, the laparoscopic sleeve gastrectomy (LSG; the stomach is divided vertically to reduce its size to about 25%, it is not reversible and it leaves the pylorus intact with a relatively quick performance through a laparoscope [7]. Several studies had shown that LSG was associated with significant weight loss, remissions and/or improvements in obesity associated co-morbidities (e.g., diabetes mellitus; DM and hypertension; HTN), reductions in mortality and in healthcare use and costs (both direct and indirect) [8,9].

In Palestine, which has developing surgical settings, there is no documented study that shows the efficacy of bariatric surgery in general and LSG in particular. This study will therefore evaluate the post-operative outcomes of LSG (mainly BMI, weight loss, DM and HTN) among obesity patients with and/or without associated co-morbidities in the Northern West Bank.

## Methods

### Study design, settings and population

A retrospective records review was conducted. The study population was all patients who had undergone LSG since the year 2010 in the Arab specialized hospital in Nablus city and the Palestinian Red Crescent society hospital in Tulkarem city, Palestine. Patients who had undergone repeated bariatric surgeries of any type, pregnant and/or lactating women in the previous 6 months were excluded as these conditions could have interfered with the study outcomes.

### Ethical and administrative considerations

The study was approved by the Institutional Review Board (IRB) and the scientific research committee of An-Najah National University-Faculty of Medicine and Health Sciences. Permissions to conduct the study in both hospitals have been obtained from the medical and administrative managers of each hospital. Subjects were invited by phone calls to participate in the study. Standard, written and same explanatory information about the study were delivered to the subjects after just answering the phone call. This information included details about aim, importance, confidentiality and anonymity of the information with optional/voluntary participation. Those who agreed to participate, met the inclusion criteria, accessible and gave their verbal consent were included in the study.

### Data collection

In the first phase, patients’ phone numbers were obtained from the hospital and were given to the researcher by the responsible surgeons. All patients (N = 36) were invited by phone call to participate and asked for their approval to review their medical records. For those who were accessible, agreed to participate and gave their verbal consent, their hospital medical records were reviewed. In overall, we were able to recruit 30 subjects with a response rate of 83.3% as 3 subjects refused to participate, and other 3 subjects were inaccessible and therefore were excluded from the study. All the finally recruited subjects (n = 30) met the study inclusion criteria.

From the medical records (first stage), the collected information included some pre-operative required information and measures (see results). In the second stage, participants were invited again by phone calls to participate. If agreed and gave their verbal consent again (for ethical consideration and quality control purposes), participants were asked to self-report some required pre-operative (not found in the medical records like food habits) and post-operative study measures (see results). All those who agreed to participate in the first stage also agreed in the second stage.

The primary study outcome was the change in the BMI before and after LSG. The secondary study outcomes were obesity associated co-morbidities’ measures; HTN and DM. Briefly, the pre- and post-operative weight, height, blood pressure for all patient were obtained from medical records and patients self-report; respectively. The pre- and post-operative Fasting Blood Sugar (FBS) and Glycated Hemoglobin A1c (HbA1c) were assessed for diabetic patient only by the similar method (pre-operatively from medical records and post-operatively by patients self-report). The pre-operative measures were those taken at referral while the post-operative were those carried out by the patients (laboratory documented) at the closest time before phone interview (usually were very near to the time of phone interviews). Changes in HTN and DM medications usage were assessed through the medical records in the pre-operative stage and by patient self-report at the post-operative stage.

### Data analysis

All data has been entered and analyzed using the statistical software package SPSS (Statistical Package for the Social Sciences) version 16 [10]. The main study outcomes were tested for normality using Shapiro Wilk significant test. For the differences between pre- and post-operative continuous outcomes, paired samples t-test and Wilcoxon test were used to analyze the differences between normally distributed and non-normally distributed variables; respectively. One-way ANOVA was used to test for the association in the mean differences of the main study outcome (post-operative BMI) among different independent categorical variables while linear regression to test for such an association with continuous independent variables. Multivariate linear regression model was developed for the main study outcome (post-operative BMI). Variables were entered in the model if they showed a significant *P*-value of <0.05 in the univariate analysis. *P-*value of less than 0.05 was considered as statistically significant.

## Results

### Description of the study participants

During the study period (January to March, 2012), we were able to investigate 30 patients. The mean ± standard deviation (SD) of the post-operative follow-up time was 7.16 ± 5.05 months. Only three patients had DM and six had hypertension. Of the overall (30 patients), nearly equal percentages of patients were from each hospital (47% from Arab specialized hospital and 53% from Palestinian Red Crescent Society hospital in Tulkarem).

As presented in Table 1, the mean age (SD) of the study population was 34 (10.7) years. Of the 30 patients, nearly 67% (n = 20) were females. Those who were not married (single, divorced and widow) constituted nearly 33% from the whole study population. We merged divorced and widow patients in the “single” category as we found small number in each (only one case each). Almost 44% (n = 13) out of 30 patients were housewives.

**Table 1:** Socio-demographic characteristics of the study population (N = 30)

**Variable**	n (%)*
Age (year)	34.06 ± 10.71^**^
Gender	
-Male	10 (33.3)
-Female	20 (66.7)
Occupation	
-Housewife	13 (43.3)
-Nurse	4 (13.3)
-Employed	7 (23.3)
-Unemployed	6 (20)
Residence	
-Tulkarem	6 (20)
-Nablus	14 (46.7)
-Jenin	5 (16.7)
-Ramallah	4 (13.3)
-Gaza	1 (3.3)
Marital status	
-Not married	10 (33.3)
-Married	20 (66.7)
Monthly family income (NIS)^§^	
-< 2500	8 (30)
-> 2500	22 (70)

*Data is presented as frequency (percent) from the total study population (N = 30).

**Mean ± standard deviation. ^§^NIS: New Israel Shekel.

### Distribution of the study participants by some food intake and lifestyle habits

As presented in Table 2, nearly half (56.7%) of the study participants reported taking more than three meals per day before surgery. This was changed into only 30% after surgery. The regular vegetables and fruits intakes were increased to 40% after surgery (23.3% and 16.7% before surgery; respectively). Before surgery, only 20% was practicing sport or exercise. However, this percentage increased to 40% after surgery. Never smokers represented 50% of our study population while current smokers represented only 36.7%.

**Table 2:** Distribution of the study participants by some food intake and lifestyle habits before and after (currently) the surgery (N = 30)

**Variable**	Before n (%)*	Currently n (%)*
How many main meals do you eat per day?		
-One	1 (3.3)	2 (6.7)
-Two	3 (10)	7 (23.3)
-Three	9 (30)	12 (40)
-> three	17 (56.7)	9 (30)
Vegetables intake		
-Regularly	7 (23.3)	12 (40)
-Occasionally	11 (36.7)	11 (36.7)
-Rarely	12 (40)	7 (23.3)
Fruit intake		
-Regularly	5 (16.7)	12 (40)
-Occasionally	12 (40)	13 (43.3)
-Rarely	13 (43.3)	5 (16.7)
How often you take fried food per week?		
-1-3	10 (33.3)	27 (90)
-4-6	13 (43.3)	1 (3.3)
-7-9	5 (16.7)	2 (6.7)
->9	2 (6.7)	0 (0)
Do you practice any kind of sport or exercise?		
-No	24(80)	18 (60)
-Yes	6 (20)	12 (40)
Smoking habits		
-Never smoker	-	15 (50)
-Ex-smoker	-	1 (3.3)
-Current smoker	-	11 (36.7)
-Argellah	-	3 (10)

*Data is presented as frequency (percent) from the total study population (N = 30).

### Evaluation of the study outcomes

Table 3 shows the mean differences and their 95% CI (confidence interval) for the study outcomes (weight, BMI, systolic and diastolic blood pressure). These study outcomes were shown to be normally-distributed (Shapiro Wilk significant test of normality was always > 0.05). As shown in the table, there were strong statistical-significant mean reduction in the weight by 30.11 kg and in the BMI by 10.50 kg/m^2^ (the 95%CI for the mean differences, *P*-values respectively are: 26.38-33.85, < 0.001 and 8.83-12.14, <0.001). Table 3 also shows the mean differences between the pre-and post-operative systolic and diastolic blood pressures where they also showed strong statistical-significant differences (Table 3).

**Table 3:** The mean ± SD for the pre- and post-operative measurements of the study outcomes and their paired samples t-test (N = 30)

**Variable**	Mean ± SD*	**Mean difference ± SD**	95% CI of the difference**	***P *** **value**
Weight (Kg)				
- Pre-operative	134.68 ± 27.17			
- Post-operative	104.50 ± 26.95	30.11 ± 10.01	26.38-33.85	< 0.001
Height (meter)				
- Pre-operative	1.68 ± 0.107			
- Post-operative	1.68 ± 0.107	0	-	-
BMI (kg/m^2^)				
- Pre-operative	47.23 ± 7.89			
- Post-operative	36.74 ± 7.74	10.49 ± 4.43	8.83-12.14	< 0.001
Systole (mg Hg)				
- Pre-operative	131.13 ± 16.84			
- Post-operative	117.83 ± 7.39	13.33 ± 16.66	7.07-19.52	< 0.001
Diastole (mg Hg)				
- Pre-operative	84.73 ± 11.90			
- Post-operative	75.83 ± 7.88	8.90 ± 12.84	4.10-13.69	< 0.001

*SD: Standard deviation. **95% CI: 95% confidence interval of the mean difference; BMI: Body mass index.

In this study, however, there were only three patients with clinically diagnosed diabetes mellitus. Their pre- and post-operative FBS and HbA1c measures are presented in Table 4 below. The means (SDs) reductions in the FBS and HbA1c were 82.00 ± 22.70 mg/dl and 1.90 ± 0.78%; respectively (*P*-values = 0.109). It should also be noted that two of those DM patients were on a once-daily dose of 850 mg metformin and after two months of operation they totally stopped their drug. The other one was on a once-daily 3 mg glimepiride dose and after one and half months of operation he decreased the dose to 1 mg once-daily.

**Table 4:** Pre- and post-operative measurements for the diabetes mellitus patients (n = 3) and the hypertensive patients (n = 6)

**Variable**	Mean ± SD*	Range**	**Mean difference ± SD**	***P *** **value**^**§**^
Diabetes mellitus patients (n = 3)				
FBS (mg/dl)				
- Pre-operative	172.32 ± 25.42	50 (200–150)		
- Post-operative	90.33 ± 17.03	30 (110–80)	82.00 ± 22.70	0.109
HbA1c (%)				
- Pre-operative	6.76 ± 1.11	2.10 (7.60-5.50)		
- Post-operative	4.86 ± 1.17	2.20 (6.20-4)	1.90 ± 0.78	0.109
Hypertensive patients (n = 6)				
Systole (mm Hg)				
- Pre-operative	146.67 ± 5.16	10 (150–140)		
- Post-operative	119.17 ± 11.14	30 (140–110)	27.50 ± 9.87	0.026
Diastole (mm Hg)				
- Pre-operative	95.00 ±5.47	10 (100–90)		
- Post-operative	76.66 ± 9.83	25 (90–65)	18.33 ± 13.66	0.042

*SD, standard deviation; FBS, fasting blood sugar; mg/dl, milligram/deciliter; HbA1c, glycated hemoglobin. **Range (Maximum-Minimum).^**§**^*P*-value of Wilcoxon test.

On the other side, there were only six patients with clinically diagnosed hypertension. Their pre- and post-operative blood pressures are presented in Table 4. Results showed that after LSG, the mean ± SD reduction in their systolic and diastolic blood pressures were 27.50 ± 9.87 (*P*-value = 0.026) and 18.33 ± 13.66 (P-value = 0.042); respectively.

### Association between the main study outcome (BMI) and other independent variables

As mentioned previously, the main study outcome was post-operative BMI. For this, we have evaluated the effect of different study independent variables on this study outcome (dependant variable). Indeed, we used one-way ANOVA analysis to test for the association in the mean differences of the post-operative BMI among different independent variables with more than two categories (including the food and lifestyles habits before and after operation). However, simple linear regression analysis was used to test for the association between the post-operative BMI and the independent variables with; (1) two categories and (2) continuous independent variables (age, post-operative time). The analysis did not reveal any significant associations (*P* >0.05) except for marital status (not-married/married; positive association), age (positive association), and practice any kind of sport or exercise currently (no/yes; strong negative association) variables. Table 5, shows this simple association between the post-operative BMI and the independent variables that found to be significant in univariate analysis.

**Table 5:** **The simple associations between the main study outcome (post-operative BMI) and marital status, practice of sport or exercise currently and age variables (N = 30)**^**§**^

	**Post-operative BMI**			
Independent variables	B	SE	Beta	*P-*value (95% CI for B)
Age (years)	0.28	0.13	0.39	0.033 (0.02-0.54)*
Marital status (not married/married)	6.88	2.76	0.43	0.019 (1.22-12.54)*
Practice any kind of sport or exercise currently (no/yes)	−8.89	2.41	−0.57	0.001 (−13.83- -3.95)*

^**§**^Simple linear regression was used. SE, standard error; B, Unstandardized regression coefficient; Beta, Standardized regression coefficient; CI, confidence interval; BMI, body mass index.*Statistically significant (p <0.05).

**Figure 1:**
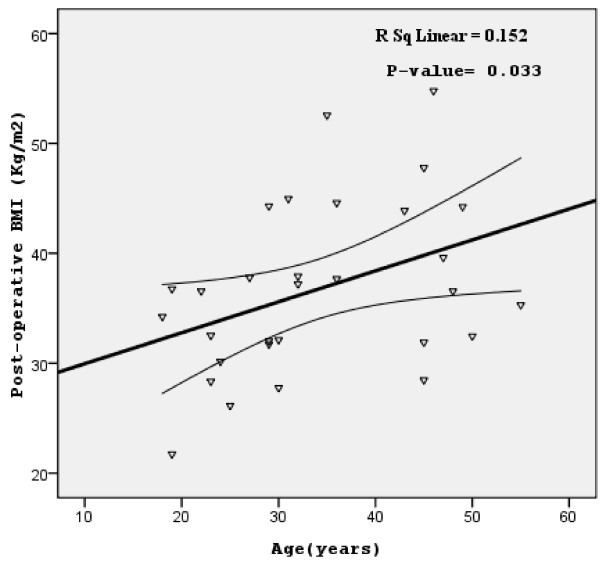
**The relationship between the post-operative BMI (main study outcome) and the participants’ age.** Scatter-plot and regression line with 95% confidence intervals of the mean are shown. BMI, body mass index [(weight (kilogram) divided by the square of height (meter)].

**Figure 2:**
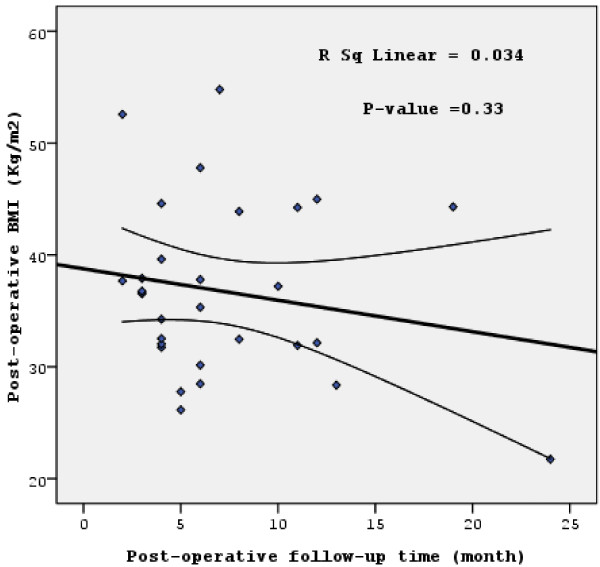
**The relationship between the post-operative BMI (main study outcome) and the participants’ post-operative follow-up time.** Scatter-plot and regression line with 95% confidence intervals of the mean are shown. BMI, body mass index [(weight (kilogram) divided by the square of height (meter)].

Figure 1 represents the relationship between the post-operative BMI and age while Figure 2 shows the relationship with the post-operative follow-up time. As shown in Figure 1, the age was positively statistically-significant with post-operative BMI (*P* = 0.033; R^2^ linear = 15.2%). On the other hand, post-operative follow-up time failed to reach a statistically-significant correlation with the post-operative BMI (although a general negative correlation was observed, *P* = 0.33*;* R^2^ linear = 3.4%; Figure 2).

### Multivariate analysis for the main study outcome (post-operative BMI)

Based on the univariate associations, we developed a multivariate linear regression model for the post-operative BMI outcome variable. Variables entered in the model if they showed significant *P*-value of less than 0.05 in univariate analysis. Table 6, however, represents the multivariate linear regression model for the post-operative BMI. Only practicing any kind of sport or exercise currently (after surgery) remained significant (i.e., not practicing sport or exercise currently were more likely to have higher BMI index compared to those practicing sport or exercise). Furthermore, this negative association was represented in Table 5 also where the one-way ANOVA showed that the post-operative BMI mean (SD) among those who did not report practicing sport or exercise was 40.3 (7.3) kg/m^2^ while it was 31.4 (4.8) kg/m^2^ among those practicing sport or exercise.

**Table 6:** **Multivariate linear regression model for the association of the main study outcome (post-operative BMI) with marital status, practice of sport or exercise and age variables (N = 30)**^**§**^

	**Post-operative BMI**			
Independent variables	B	SE	Beta	*P-*value (95% CI for B)
Age (years)	0.17	0.12	0.23	0.18 (−0.83-0.42)
Marital status (not married/married)	1.84	2.97	0.11	0.54 (−4.28-7.95)
Practice any kind of sport or exercise currently (no/yes)	−7.33	2.60	−0.47	0.009 (−12.68- -1.98)*

^**§**^Variables entered in the model are those with a *P-* value <0.05 in univariate analysis. SE, standard error; B, Unstandardized regression coefficient; Beta, Standardized regression coefficient; CI, confidence interval; BMI, body mass index [(weight (kilogram) divided by the square of height (meter)]. *Statistically significant (p <0.05). Enter regression method was used.

## Discussion

To our knowledge, this is the first study in Palestine that evaluates the outcomes of LSG. The main study findings were that LSG showed remarkable success in both reducing BMI (main study outcome) and obesity related complications. Clearly, the study showed that there was a strong statistically significant reduction in the mean of the BMI from (mean ± SD) 47.23 ± 7.89 kg/m^2^ pre-operatively to 36.74 ± 7.74 kg/m^2^ post-operatively (95% CI for the mean difference and *P*-value; 8.83-12.14 kg/m^2^ and <0.001). Regarding weight alone, there was also a statistically significant reduction in the mean ± SD from 134.68 ± 27.17 pre-operatively to104.50 ± 26.95 kg post-operatively (95% CI for the mean difference and *P*-value; 26.38-33.85 kg and <0.001; Table 3). These results were found after a mean follow up (post-operative time) of nearly 7.2 months (SD = 5 months). LSG could therefore be considered as a significant intervention for decreasing both BMI and weight in adults. These results were in accordance with previous studies. One study for example, showed a significant reduction in the mean of BMI from 37.4 ± 6.2 to 30.1 ± 5.9 kg/m^2^ after 6 months [11]. Another study was performed among morbidly obese patient underwent LSG. The findings showed that patients who underwent LSG had a significant mean weight ± SD decrease from 161.42 ± 34.3 to 112.91 ± 25.55 kg (p < 0.01) where the mean ± SD of BMI was decreased from 57.5 ± 9.55 to 39.85 ± 7.1 kg\m^2^ (p < 0.01) after one year of follow up [6]. Another study in India showed that patients’ body weight decreased significantly from a pre-operative of 131.2 ± 21.6 kg (mean ± SD) to a post-operative of 99.4 ± 16.6 kg with a BMI decrease from 47.0 ± 7.9 to 34.7 ± 5.8 kg/m^2^ (mean ± SD) [12].

In general, studies showed that significant improvement or remission of diabetic markers was commonly observed after bariatric surgery and improvement of co-morbid conditions after LSG [13]. In our study we found a mean decrease of FBS and HbA1c among the three DM patients of 82 mg/dl and 1.9% respectively. Wilcoxon test analysis for the pre- and post-operative measures of those patients did not showed statistical significant differences (*P* = 0.109) eventually due to small sample size (n = 3) although there were clinically and biologically clear reductions in both measures (Table 4). Moreover, two DM patients in our study stop their medications after two months of the LSG while one DM patients decrease the dose to one-third. More recently in 2010, LSG and type 2 diabetes mellitus (T2DM) were studied in a review and included all studies from 2000 to 2010. In overall, they found that DM had resolved in 66.2% of the patients, improved in 26.9%, and remained stable in 13.1%. The mean decrease in FBS and hemoglobin HbA1c levels after LSG was 88.2 mg/dl and 1.7%, respectively [14]. We have reported nearly the same results among the Palestinian population of the Northern West Bank.

Regarding the blood pressure outcome, in our study there were general reductions among all participants’ systolic and diastolic blood pressure. As expected, there was a benefit from LSG regarding blood pressure. Indeed, we found a significant mean reduction in both systolic and diastolic blood pressure for the whole study population (n = 30) of about 13.33 and 8.90 mg Hg; respectively (Table 3). In our study however, we had only 6 clinically diagnosed hypertensive patients. After LSG, there was a significant mean ± SD reduction in their systolic and diastolic blood pressures of 27.50 ± 9.87 mg Hg (*P*-value < 0.026) and 18.33 ± 13.66 mg Hg (*P*-value <0.042); respectively (Table 4). These findings indicate that the benefit from LSG regarding blood pressure is more significant for those with clinically diagnosed hypertension. We found no previous study that specifically measured the mean reduction in systolic and diastolic blood pressures for hypertensive patient underwent LSG. However, after one year of follow-up, a study described the changes in the blood pressure for all participants underwent LSG. It concluded that LSG was effective for reduction of cardiovascular risk in severely obese patients at one year of follow-up. The systolic blood pressure decreased from 128.5 ± 12.9 to 113.4 ± 13.1 mg Hg (mean ± SD), whereas diastolic boold pressure decreased from 81.8. ± 9.5 to 71.9 ± 8.0 mg Hg (mean ± SD) for all patients who underwent LSG (40 participant) [15]. Our results were nearly approximating the above study findings which suggest that LSG is found again to be effective among the Palestinian population in reducing their general blood pressure. This is supported by the finding that 4 (66%) out of our 6 hypertensive patients showed improvement in their blood pressure after LSG as indicated by frequency of medication usage. One retrospective study, for example, reviewed 130 patients who underwent LSG from January 2003 to May 2004 and aimed to evaluate the results of LSG at one year in morbidly obese Korean patients. The results showed that HTN was resolved in 92.9% and improved in 100% of patients [16]. Another study was conducted in 2008 and reviewed data for 100 patients who underwent LSG at Counties Manukau District showed that, with mean age 43 years, 60% of patient who had hypertension pre-operatively (n = 45) resolved after one year post-operatively [17]. It should be noted however that, the small number of diabetic and hypertensive patients in our study could be due to the small sample size from one hand and to the relatively young mean (SD) age of about 34 (10.7) for our participants from the other hand.

As mentioned previously, the main study outcome was post-operative BMI. For this, we have evaluated the effect of different study independent variables on this study outcome (dependant variable). Although the association between the main study outcome (post-operative BMI) and the post-operative time (Figure 2) failed to reach a statistically significant result (*P* = 0.33), a negative association was observed with an R^2^ linear of about 3.4%. We did not find a study that correlated the post-operative BMI with the post-operative follow-up time. However, our finding could be explained by that as long as the time pass after LSG surgery, the benefit is increased through the decrease in the post-operative BMI [18].

The other univariate one-way ANOVA and simple linear regression analyses did not reveal any significant associations (*P* >0.05) except for marital status (not-married/married), age (continuous) which individually showed a positive association (*P* <0.05, Table 5 and Figure 1) and practicing any kind of sport or exercise after surgery (no/yes) that in turn showed a strong negative association (*P* <0.05). In multivariate linear regression analysis, only practicing any kind of sport or exercise after surgery (no/yes) remained statistically significant predictor of post-operative BMI with negative association (95% CI for B and the *P*-value are; -12.68- -1.98 and 0.009; i.e., not practicing sport or exercise after surgery was more likely to be associated with higher BMI index compared to those practicing sport or exercise). The explanation of these multivariate results of practicing exercise and/or sport can be explained by the effect of physical activity on consuming calories and decreasing the weight in general. For those with BMI of more than 40, some studies showed that BMI did not show significant changes with the guided physical activity program (lasted 3 months with 5-weekly sessions of about 45 minutes each) unless accompanied by dietary restriction [19]. This could explain why increasing physical activity after LSG in our study was associated with a decrease in the post-operative BMI as LSG eventually works by decreasing the size of the stomach and thus the amount of food intake.

Despite the great efforts we exerted to include more participants in our study, this study was mainly limited by the small sample size (n = 30) and nearly the short post-operative follow-up time (mean = 7 months) due to limited resources and time. This might have limited the analysis of some variables in our study as well as the generalization of our results. Therefore, further larger and long-term studies are needed to evaluate the efficacy of LSG in adults and to generate the results of this study. Another limitation of our study is that some of our pre-and post-operative measures were self-reported by the patients. This could have resulted in the recall bias where an over-and/or under-estimation could have been occurred and therefore affected some of our variables mainly when reporting food intake and lifestyles habits from the study questionnaire. However, other variables like socio-demographic, FBS, Hb1Ac, weight, height, post-operative time, systolic and diastolic blood pressures could not have been misestimated as those participants represents an informative group of patients who had undergone a major and recommended surgical operation for a strong purpose and motivation. It represents for them an important intervention to control their BMI. Therefore we belief that they know very well their personal measurements related to this operation and are on a continuous self-monitoring and observation to their physical changes and co-morbidities. In the meanwhile, the patients reported their FBS and the Hb1Ac from the closest documented laboratory test performed prior to phone interview. We have nearly equal number of participants from both hospitals; therefore the effect of the operation setting is expected to be minimal.

## Conclusions

LSG can significantly improve the BMI. It may also improve and/or resolve obesity associated co-morbidities like blood pressures and DM. We suggest that, LSG is an effective option in obese adults resulting in a significant weight loss. It could be recommended as a useful and single intervention therapy for co-morbid obesity patients who usually fail to reach and provide real benefits from a structured weight loss programs.

## Abbreviations

BMI: Body Mass Index; CI: Confidence Interval; DM: Diabetes Mellitus; FBS: Fasting Blood Sugar; HbA1c: Glycated Hemoglobin; HTN: Hypertension; LSG: Laparoscopic Sleeve Gastrectomy; SD: Standard Deviation; SE: Standard Error; T2DM: Type 2 Diabetes Mellitus.

## Competing interests

The authors’ declare that they have no competing interests.

## Authors’ contributions

HA and AD designed and coordinated the study protocol, conducted the statistical analysis and drafted the manuscript. AD collected the data from medical records and patients. AH and JH performed the surgical operations and reviewed the manuscript. All authors read and approved the final manuscript.
